# The Treatment of Burns

**Published:** 1894-03

**Authors:** J. W. Lindsey

**Affiliations:** Claysburg, Pa.


### THE TREATMENT OF BURNS.

By J. W. LINDSEY, M. D., CLAYSBURG, PA.

I have no doubt that your method of treatment of burns advocated in the December issue of the *College and Clinical Record* would be a good one in the hospital or in a rich family, but where you have poor people, and are at a great distance from hospitals, the object is to do good work skillfully and with the smallest expense possible. Allow me, therefore, to present what is partially a new as well as a very successful mode of treating burns.

I was recently called on about two o'clock P. M. by a brother of a young man aged eighteen years, who lived about five miles from here. He stated to me that his brother had been making a fire in an old Dutch oven and used oil, and when he set the can away, possibly eight or ten feet from the oven, and lighted it, it ignited and exploded, pouring the oil almost all over the entire surface of his limbs from the knees to his umbilicus and between his thighs, then leaving a space of about six or seven inches and involving the whole breast. His arms and hands were burned up to the shoulders, and his right hand was so badly drawn and burned that the tendons of all his fingers were jumped over the last joints. His tongue was greatly swollen and burned, so that the outer coating came off; his lips were about three times as thick as normal. The left side of the face and ear were badly burned, and the hair of his head was almost entirely burned away.

His penis and scrotum were also very badly burned, and for seven days it was necessary to use a catheter; the scrotum was swollen as large as three fists, and the penis as thick as a man's arm. He was breathing forty-eight times a minute, and when breathing he would whistle so that you could hear him twenty-five to thirty feet away.

I gave him one-fourth grain of sulphate morphia every three or four hours until asleep. I reduced the tendons of his hand to their normal places and dressed him with the following mixture:

℞ Sodii bicarbonatis.................................℥ xvj.

Ol. lini.........................................O ij.

This made a heavy paste which I left on until the next day. I then used:

℞ Sodii bicarb.......................................℥ xvj.

Acidi carbolici.................................ʒ j.

Ol. lini.........................................O ij.

M. Fiat unguentum.

Sig.—Spread over the entire surface of the burn.

I kept this on for three or four days.

In a few days I dissected all the burned tissues away except on the penis and scrotum, which I left for about ten days or two weeks, when it peeled off itself.

I then dressed him with the following ointment:

℞ Iodoformi.........................................℥ iv.
Zinci oxidi......................................ʒ iv.
Ol. lini..........................................O j.
M. Fiat unguentum.
Sig.—Apply on muslin over the affected surfaces.

After a week I substituted the following:

℞ Balm of Gilead buds juice, ........ O j.

Sheep's tallow ........ ℥ iv.

Rosin ........ ℥ j.

Beeswax ........ ℥ ss.

M. Fiat unguentum.

Sig.—Apply on muslin cloths once or twice a day.

On the twentieth day I made passive motion of the elbows and fingers, and so on until the thirty-first day, when all was healed. I did no skin grafting and had no trouble, as all the healing was in good condition from the beginning. Granulations were set up very early and continued in a healthy condition.

He is not crippled in any manner except the weakness of his breast, but the muscles and mammary glands were all burned away and as a consequence he has not much strength. As for marks, there are very few, none that show on the face or ear; and on his hands a slight redness is discernible, but there are no scars.—*The C. and C. Record.*

